# Direct and Indirect Impacts of Infestation of Tomato Plant by *Myzus persicae* (Hemiptera: Aphididae) on *Bemisia tabaci* (Hemiptera: Aleyrodidae)

**DOI:** 10.1371/journal.pone.0094310

**Published:** 2014-04-07

**Authors:** Xiao-Ling Tan, Su Wang, James Ridsdill-Smith, Tong-Xian Liu

**Affiliations:** 1 State Key Laboratory of Crop Stress Biology for the Arid Areas, and the Key Laboratory of Crop Pest Management on the Losses Plateau of Ministry of Agriculture, Northwest A&F University, Yangling, Shaanxi, China; 2 Institute of Plant and Environment Protection, Beijing Academy of Agriculture and Forestry Sciences, Beijing, China; 3 School of Animal Biology, The University of Western Australia (M092), Crawley, WA, Australia; Institute of Vegetables and Flowers, Chinese Academy of Agricultural Science, China

## Abstract

The impacts of infestation by the green peach aphid (*Myzus persicae*) on sweetpotato whitefly (*Bemisia tabaci*) settling on tomato were determined in seven separate experiments with whole plants and with detached leaves through manipulation of four factors: durations of aphid infestation, density of aphids, intervals between aphid removal after different durations of infestation and the time of whitefly release, and leaf positions on the plants. The results demonstrated that *B. tabaci* preferred to settle on the plant leaves that had not been infested by aphids when they had a choice. The plant leaves on which aphids were still present (direct effect) had fewer whiteflies than those previously infested by aphids (indirect effect). The whiteflies were able to settle on the plant which aphids had previously infested, and also could settle on leaves with aphids if no uninfested plants were available. Tests of direct factors revealed that duration of aphid infestation had a stronger effect on whitefly landing preference than aphid density; whitefly preference was the least when 20 aphids fed on the leaves for 72 h. Tests of indirect effects revealed that the major factor that affected whitefly preference for a host plant was the interval between the time of aphid removal after infestation and the time of whitefly release. The importance of the four factors that affected the induced plant defense against whiteflies can be arranged in the following order: time intervals between aphid removal and whitefly release > durations of aphid infestation > density of aphids > leaf positions on the plants. In conclusion, the density of aphid infestation and time for which they were feeding influenced the production of induced compounds by tomatoes, the whitefly responses to the plants, and reduced interspecific competition.

## Introduction

The sweetpotato whitefly, *Bemisia tabaci* (Gennadius), is an exotic insect pest in China and causes severe damage by direct feeding and transmitting viruses on various vegetables, ornamental and field crops [1], [2]. Many appropriate strategies have been promoted to suppress *B. tabaci* taking into consideration their characteristics of high plasticity adaptation to the environment, wide host plant range and strong pesticide resistance [3], [4], [5], [6].

As an invasive species, *B. tabaci* faces competition from native phytophagous arthropods which are in the same niche and share a similar food range. The competition may be attributed to the contest for food resources and space for reproduction [7]. Previous work has indicated that competition exists between *B. tabaci* and *Trialeurodes vaporariorum* (Westwood) on greenhouse-grown vegetables and ornamentals [3]. Colonization of *B. tabaci* may negatively influence the development and survival of the cabbage looper, *Trichoplusia ni* (Hübner) and the vegetable leafminer, *Liriomyza sativae* (Blanchard) [8], [9]. Likewise, infestation of *B. tabaci* can decrease the population density of *M. persicae* [6] and the two-spotted spider mite, *Tetranychus urticae* (Koch) [10]. In addition, the occurrence of other herbivores might influence the colonization of *B. tabaci* via host plant induced defense reactions [11], [12], [13].

The competition among herbivores may rely mostly on damage-induced reactions in plants [14], [15]. It has been documented in over 100 plant species that previous insect infestation promotes resistance of plants against herbivores [16]. This kind of induced response to herbivores includes not only production of direct defenses, such as toxins and other plant defensive traits that are only expressed in response to herbivores, but also involved the enhanced attraction of predators [17], [18]. For instance, chewing caterpillars induced the synthesis of proteinase inhibitors and accumulation of other chemicals in tomato plants that make life difficult for chewing insects on those branches of the attacked plant [16], [19]. Similarly, following attack by *B. tabaci*, collard and tomato produce pathogenesis related (PR) proteins, which negatively affect the colonization process of conspecific and heterospecific competitors [20]. Some studies show that induced resistance can be attributable to changes in the emission of volatile compounds by plants previously infested by insects [8], [21], [22], [23]. For example, feeding of *B. tabaci* induced a defense in tobacco plants against *M. persicae* [6]. Also, infestation of the beet armyworm, *Spodoptera exigua* (Hübner), strongly induces volatile emission; whereas infestation with *B. tabaci* (biotype B) does not induce volatile emissions in cotton [24].

Available information on plant induced responses to herbivores is rarely focused on phloem-feeding insects, such as whiteflies or aphids [12], [25], [26]. Recently, more studies have focused on the defense of plants to phloem-feeding insects, especially against aphids [27]–[30]. Unfortunately, little is known about responses of whitefly to plants previously attacked by other arthropods or by other inducer factors [31].

Evidence indicates that induced defenses may have lower resource allocation costs than constitutive defense traits, and reduced the plant's energy expenditure by allowing it to invest in defense only when necessary, and to avoid costly allocations to defense when herbivores are not present. Inducible defenses may be particularly effective if the initial herbivory is unpredictable, but subsequent herbivory is likely [11], [17]. Frequently, generalist herbivores, such as *B. tabaci*, are more affected by plant defense responses than specialists [11], [32]. The phloem-feeding insect *M. persicae* is a generalist species on its host plant and has evolved to survive on a nutritionally imbalanced diet of phloem sap, compared with chewing insects [33], [34]. More importantly, insects from different feeding guilds tend to elicit distinct patterns of gene expression whereas attackers from the same guild, like *M. persicae* and *B. tabaci*, evoke very similar responses [17], [35]. Previous studies demonstrated that *M. persicae* feeding induces expression of PR genes and other transcripts associated with salicylic acid (SA)-mediated signaling, similar to the host responses observed with pathogens or SA treatment [36]. SA-mediated signaling defenses may have evolved as a relatively nonspecific strategy to deter a large variety of different herbivores. And some defense proteins do not follow the principle of being either herbivore induced or pathogen induced [17]; therefore, feeding of *M. persicae* may induce defense against the whitefly on tomato plant. Both SA and JA dependent pathways have been demonstrated to be activated in tomato in response to feeding by *Macrosiphum euphorbiae* (Thomas) [35], [37]. It is likely that *M. persicae* and *B. tabaci* exist simultaneously on the same tomato plant, and that plant–mediated interactions or competition between the two species may occur. We used two phloem-feeding species, *B. tabaci* and *M. persicae*, on the same host plant to reveal influences of induced defense by preinfestation of the latter species on the former.

Recently, more studies have focused on the defense of plants to phloem-feeding arthropods, including whiteflies interfere with indirect plant defense against spider mites [4], volatile communication in plant-aphid interactions [18], previous infestation of *M. persicae* on the setting behavior and reproduction of the same aphid species [30], transcriptomics and functional genomics of plant defense induction by phloem-feeding insects [35], and the suppression of the induction of the salicylic acid and jasmonic acid signaling routes involved in induced plant defenses by an invasive spider mite *Tetranychus evansi* Baker & Pritchard [38]. In a previous study [6], we determined the induced defense by the feeding of one phloem-feeding insect, *B. tabaci*, against another, *M. persicae*, and found that the feeding of *B. tabaci* on tobacco induced defenses against *M. persicae* both locally and systemically. However, many questions are not well answered, including what is the defense effect of the plant on the newcomer after a primary feeder is removed, how long the defense lasts, and whether the induced defense is density-dependent or not.

In this study, we used the same two phloem-feeding insects, *B. tabaci* and *M. persicae*, as we did in the previous study [6]. Our specific objectives included: (1) to determine whether infestation by *M. persicae* could induce defense of tomato plants to *B. tabaci*; (2) how do whiteflies respond in choice and no-choice experiments between plants with and those with no aphids; (3) to determine the importance of different levels of four factors: (A) durations of aphid infestation, (B) density of aphids, (C) interval between aphid removal and the time of whitefly release, and (D) positions of the leaves located on the plants; and (4) to compare the highest and lowest responses of whiteflies across the four factors to react to uninfested control plants.

## Materials and Methods

### Host Plant and Insects

Tomato, *Solanum lycopersicum* L. (var. Baofen-F1, 2008, Changfeng Institute of Vegetable, Lintong, Xi'an, Shaanxi, China) was used as the host plant. The tomato was cultured in plastic trays (50.0×25.0×15.0 cm), eight plants per tray. Seedlings, 4–5 cm in height, were transplanted into plastic pots (20 cm in depth and 15 cm in diameter) and were placed in clean cages (60 × 60 × 60 cm; plastic frame, screened with 120 mesh nylon yarn net). Plants used in all experiments were approximately 30 cm in height with 5–7 true and fully expanded leaves. The experiments were conducted in walk-in chambers at 25±2°C, 65±5% RH, and a photoperiod of 16∶8 (L: D) h with artificial lighting at 3500 lx.

*Myzus persicae* (>15,000) were collected from pepper plants (*Capsicum annuum* L.) (var. Jingyuan New Prince, provided by Qing County Modern Agricultural Technology Promotion Center, Hebei Province) in a greenhouse on the campus of Northwest A&F University, Yangling, Shaanxi (116°22′42″ E and 39°59′58″ N) in March 2011. The aphids were maintained on the same pepper variety under the laboratory conditions as described above. The 4th instar nymphs of aphids were used after they were reared for more than five generations on peper plants. When some of these aphids were moved to tomato plants, they were reared for several generations. There was no evidence that the host switch influenced their feeding especially since they only fed for a maximum of 72 h on the tomatoes.

*Bemisia tabaci* B (507 males and 631 females) were collected from tomato plants in a greenhouse near the campus of Northwest A&F University, Yangling, Shaanxi, China, and were cultured on tomato with similar conditions as described for aphids from March to April, 2011. The whiteflies were used in all experiments after they had been reared on the plants for more than five generations.

### Bioassays

There were seven experiments, four with whole plants and three with detached leaves. In all cases aphids were allowed to feed and at some point removed. Whiteflies were added when aphid were present (direct effects) or after they were removed (indirect effects). For the whole plants there were choice and no-choice experiments, and for the detached leaves whitefly were often applied to factorial levels of treatments of aphid infestations. Control treatments with no aphids were used with whole plants and in the final (Exp. 7) comparing leaves with factorial treatments with the highest and lowest whitefly responses with a control leaf.

The whitefly adults were collected from the insectary colony using an aspirator, and then were blown onto the plants through the door of the screen cage. Numbers of whitefly adults that landed on the leaves were counted and recorded at 30-min intervals for eight hours. The landing and taking-off behaviour of the whitefly adults were recorded using a HD-Digital video camera (FZ-1, Sony Corporation, Tokyo, Japan) attached on the top of the cage. When we designed the experiment, we had anticipated that the number of whiteflies would remain relatively constant for 8 h within each treatment. However, we observed that the number of whiteflies settling on the leaf surfaces frequently increased during the 8 h, and then we decided to use the percentage of the whiteflies introduced and that had settled at 8 h as the measure to be analyzed (there were 40 replicates of each treatment). A scatter plot joined by straight lines was used to show differences in whitefly behavior in the different treatments.

#### Effects of *M. persicae* Infestation on *B. tabaci* Response

There were four experiments with whole plants (Exps. 1–4), two direct and two indirect experiments, and each had a choice and a no-choice experiment. For the direct effect experiments (Exps. 1 and 2), the aphids were present on the plants (3–5 true leaves) when whiteflies were released; and for the indirect effect experiments (Exps. 3 and 4), the aphids fed on the plants for 24 h and were then removed before introducing *B. tabaci* adults. In the direct-choice effect experiment (Exp. 1), 40 newly emerged whitefly adults (20 pairs of males and females) were offered a choice of two tomato plants in a cage (50×50×50 cm, wood-framed with 100 mesh of plastic screen); one plant was infested with 40 aphids (fourth-instar nymphs) for 24 h, while the control plant was not infested by aphids. In the direct no-choice effect experiment (Exp. 2), two tomato plants were separated placed in a cage; one plant was infested with 40 aphids, and the aphids remained on the plant until the end of the experiment; another plant was not infested by aphids. Twenty newly emerged whitefly adults were separately released into each of the two cages. Each experiment was replicated 40 times.

In the indirect effect test, the experimental design was exactly the same except that 40 aphids were introduced on the aphid-treatment plants in both the choice test (indirect-choice) (Exp. 3) and the no-choice tests (indirect-no-choice) (Exp. 4), and the aphids were then removed after feeding on the leaves for 24 h, immediately before the whitefly adults were introduced to the cages.

#### Effect of *M. persicae* Infestation Duration, Density, Interval Duration after Aphid Feeding and Leaf Position on *B. tabaci*

Two experiments using detached leaves were conducted: a direct aphid effect experiment (Exp. 5) and an indirect effect experiment (Exp. 6).

*Direct effect experiment*. In this experiment (Exp. 5), the direct effects of aphids on whiteflies were determined by the treatments of aphids in a 3×3 factorial experimental design (Table 1): 24, 48 or 72 h of aphid infestation at 20, 50 or 80 aphids/leaf. The aphids were separately released on a middle leaf of a plant, and the leaf was placed in a zip-lock bag with numerous needle-holes (≈0.5 mm in diameter) for ventilation. The aphids were allowed to feed on the leaf for 24, 48 or 72 h, and the bag covering the leaf was removed. The leaf was detached, the petiole was inserted in a glass bottle filled with water and was placed in a plastic cage (13×13×30 cm; 120 mesh nylon yarn net door on one side). Twenty newly emerged whitefly adults (10 pairs of males and females) were introduced into the cage. Each treatment had 10 replicates, repeated until 40 replicates were completed.

**Table 1:** The different treatments with three infestation durations (A_1_ = 24 h, A_2_ = 48 h, and A_3_ = 72 h), three aphid densities (B_1_ = 20 aphids/leaf, B_2_ = 50 aphids/leaf, and B_3_ = 80 aphids/leaf) in direct influence assays.

No.	Infestation duration(h)	Aphids density (aphids/leaf)
1	A_1_	B_1_
2	A_1_	B_2_
3	A_1_	B_3_
4	A_2_	B_1_
5	A_2_	B_2_
6	A_2_	B_3_
7	A_3_	B_1_
8	A_3_	B_2_
9	A_3_	B_3_

*Indirect effect experiment*. A more complex experiment with four factors that affect the whitefly infestation was conducted (Exp. 6). The four factors included: durations of aphid infestation (24, 48 or 72 h), aphid density (20, 50 or 80 aphids/leaf), interval between the time of aphid removal after infestation and the time of whitefly adult release (0, 24 or 48 h), and three leaves in the middle part of a plant (one leaf was infested with aphids), or the leaf above or the one below the aphid infested leaf, which were not infested with aphids. The aphids (20, 50 or 80/leaf) were released on a middle leaf of the three selected leaves, and this leaf was covered with a zip-lock bag with numerous needle-holes (≈0.5 mm in diameter) for ventilation. The aphids were allowed to feed on the leaf for 24, 48 or 72 h, and then were removed. The three leaves were detached from the plant, 0, 24 or 48 h after aphids had been removed. The middle leaf, the one above or the one below the middle leaf were each arranged in a glass bottle filled with water placed in a plastic cage (13×13×30 cm; 120 mesh nylon yarn net), and 20 new emerged whitefly adult females were released into the cage. Numbers of *B. tabaci* that landed on the treated leaf, or the upper or the lower one were recorded as described above. Each treatment was tested for 10 replicates, repeated until 40 replicates were completed. Because there were a total of 81 treatments (3×3×3×3 or 3^4^) as designed above, a L_9_ (3^4^) orthogonal design was used, and the number of treatments was reduced from 81 to 9 (Table 2).

**Table 2:** An orthogonal experimental design with three infestation durations (A_1_ = 24 h, A_2_ = 48 h, and A_3_ = 72 h), three aphid densities (B_1_ = 20 aphids/leaf, B_2_ = 50 aphids/leaf, and B_3_ = 80 aphids/leaf), three time intervals (C_1_ = 0 h, C_2_ = 24 h, and C_3_ = 48 h), and three leaves (D_1_  =  the leaf above the aphid-infested leaf, D_2_  =  the aphid-infested leaf, and D_3_  =  the leaf below the aphid-infested leaf).

No.	A. Infestation duration (h)	B. Aphid density (aphids/leaf)	C. Time Intervals (h)	D. Leaves
1	A_1_	B_1_	C_1_	D_1_
2	A_1_	B_2_	C_2_	D_2_
3	A_1_	B_3_	C_3_	D_3_
4	A_2_	B_1_	C_2_	D_3_
5	A_2_	B_2_	C_3_	D_1_
6	A_2_	B_3_	C_1_	D_2_
7	A_3_	B_1_	C_3_	D_2_
8	A_3_	B_2_	C_1_	D_3_
9	A_3_	B_3_	C_2_	D_1_

The highest and lowest responses of B. tabaci in the direct and indirect treatments with aphid infestations against a control plant with no aphid infestation. In the last experiment (Exp. 7), two treatments from the direct experiment (Exp. 5) and two treatments from the indirect experiment (Exp. 6) were selected, and the responses were compared with a control treatment: in each case the treatment attracting the most whiteflies (highest whitefly response) and fewest (lowest response) were selected. From Exp. 5, the highest response treatment was 50 aphids feeding on the leaf for 24 h, and the lowest response treatment was 20 aphids feeding on the leaf for 72 h. From Exp. 6, the highest response treatment was that 50 aphids/leaf fed on the middle leaf for 24 h and then the whitefly adults were released on the lower leaf 48 h after aphid removal; and the lowest response treatment was that 80 aphids/leaf fed on the middle leaf for 72 h, and then the whitefly adults were released on the target leaf 24 h after aphid removal. The control treatment was a leaf from a clean plant without aphid infestation. Number of whitefly adults that landed on each leaf was recorded as described above. Ten replicates of each treatment were tested, repeated until 40 replicates were completed.

### Data Analysis

Percentages (the ratios of the whitefly number of landing on the plant to the total number of releasing in the cage) of the whiteflies that landed on plant leaves after 8 h were arcsine-square-root-transformed before analysis, and the means for the last observation points were subjected to *t*-test, was used to analyze the percentage of whiteflies landing on aphid infested leaves, and plants without aphid infestation in choice and no-choice tests (SPSS version 17.0, 2010; SPSS, Chicago, IL, USA). Factorial ANOVA were used to analyze the last obsevation point (8 h) of percentages of whitefly adults which landed on the plant leaves with different aphid densities and different durations of infestation. Fisher's positive Tukey test was used to compare the mean percentages of whiteflies that landed on the leaves when a significant effect was found (*P*<0.05). In indirect effect experiments of orthogonal design, factorial ANOVA was used to analyze the last observation data among the effects of the four factors with an orthogonal experiment design (Tables 2 and 3).

**Table 3:** Comparison of single factor approximation values in each independent factor of percentage of *B. tabaci* in the four factors in an orthogonal experimental design.

Source		Mean ± SE*
A. Infestation duration	24 h	39.8±0.7a
	48 h	32.6±0.7b
	72 h	29.2±0.7c
B. Aphid density	20 aphids/leaf	37.7±0.7a
	50 aphids/leaf	39.1±0.7a
	80 aphids/leaf	28.4±0.7b
C. Time intervals	0 h	25.9±0.7b
	24 h	24.8±0.7b
	48 h	35.4±0.7a
D. Leaves	Aphid-treated leaf	30.7±0.7b
	Higher leaf	32.9±0.7a
	Lower leaf	34.2±0.7a

*The means (±SE) followed by the different small letters in the same source (subcolumn) indicate that the means are significantly different (*P* = 0.05; Fisher's positive Tukey test).

## Results

### Effects of *M. persicae* Infestation on *B. tabaci* Response

#### Direct Effect Experiment

**Figure 1:**
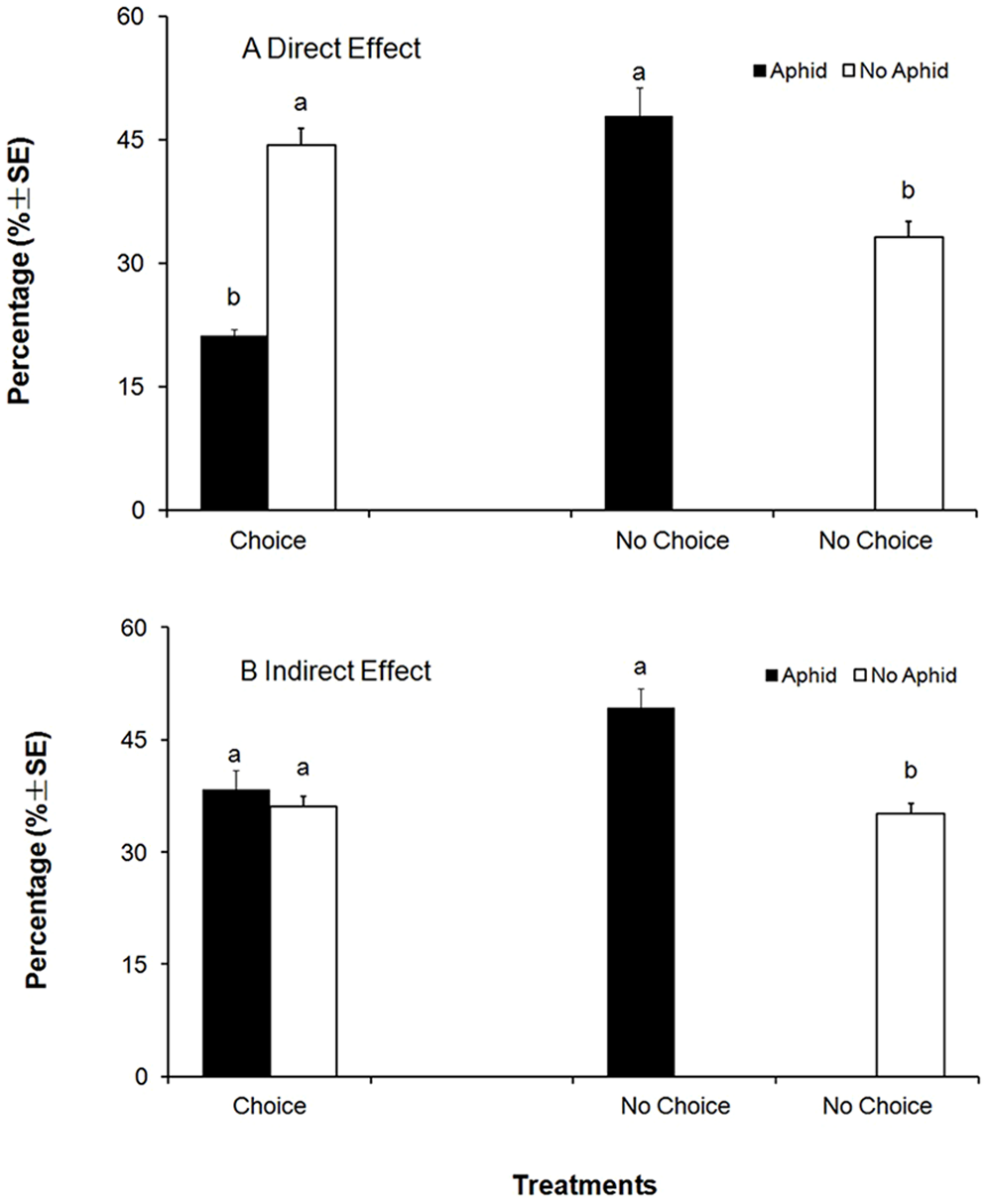
Mean percentages (±SE) of *B. tabaci* adults on treated and untreated plants. A. On the leaves with aphids (direct effects); B. On the leaves after aphids were removed (indirect effect).

**Figure 2:**
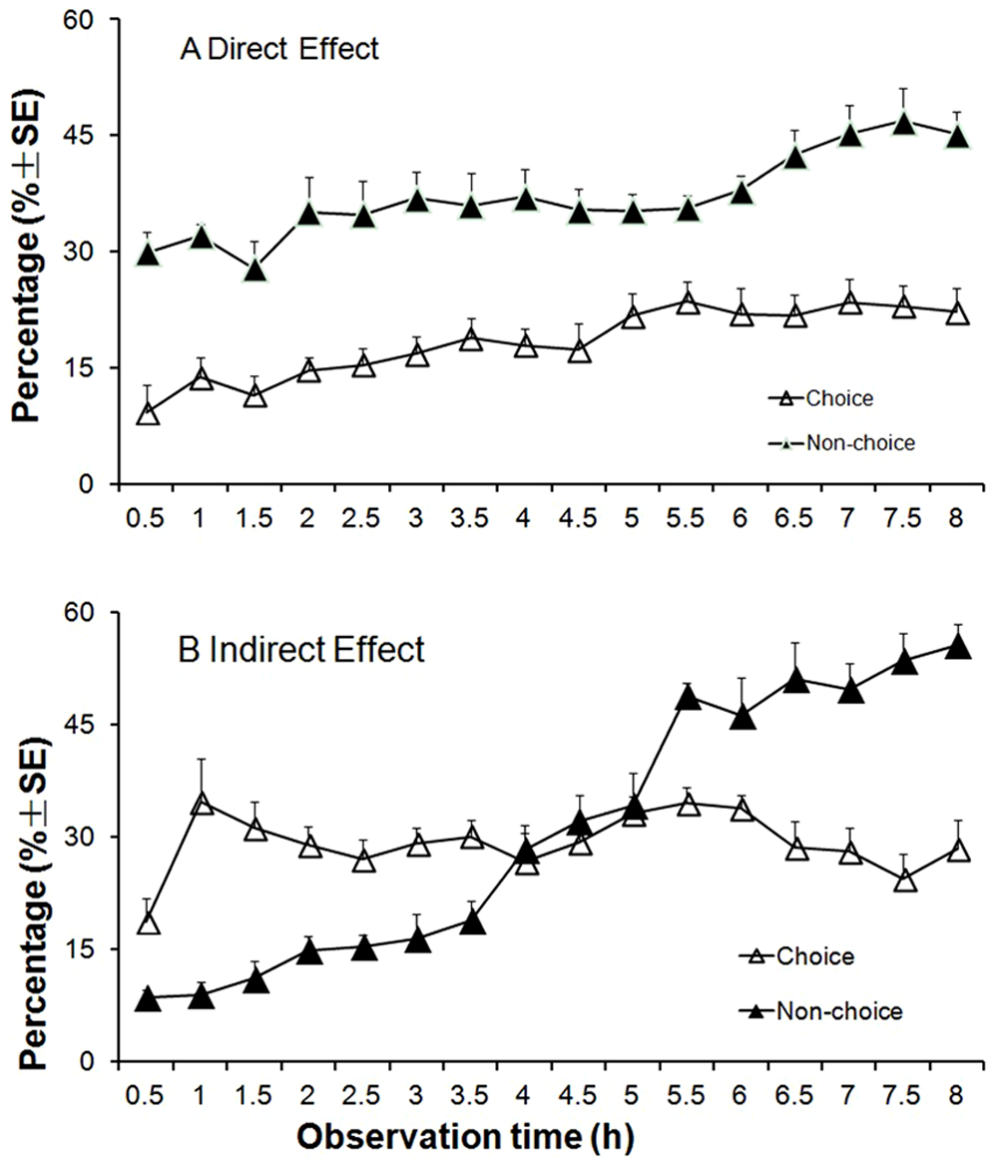
Mean percentages (±SE) of *B. tabaci* adults on treated leaves over different observation times in direct and indirect bioassays. A. On the leaves with aphids (direct effect); B. On the leaves after the aphids were removed (indirect effect).

In the choice experiment (Exp. 1), the percentages of whitefly adults that landed on the plant with aphids were significantly less than those on the plants without aphids (*t* = −18.69; df = 78; *P*<0.01) (Fig. 1A). In contrast, in the no-choice experiment (Exp. 2), more whitefly adults were found on the plants infested with aphids than those without aphids (*t* = 3.27; df = 78; *P* = 0.002) (Fig. 1A). The video footage showed that the whiteflies landed on the treated plant in no-choice test were more than that in choice test, and once whiteflies landed on the plants, they will not fly away, the average percentage of whitefly adults landing on the leaves increased over time (Fig. 2A).

#### Indirect Effect Experiment

Percentages of whitefly adults on the aphid-infested plants and those without aphids were not significantly different in the choice experiment (Exp. 3) (*t* = 0.974; df = 78; *P* = 0.333) (Fig. 1B). However, in the no-choice experiment (Exp. 4), more whitefly adults were found on the plants infested with aphids than those no-infested with aphids (*t* = 10.12; df = 78; *P* = 0.001) (Fig. 1B). In the no-choice experiment, the percentage of whitefly adults landing on the leaves increased over time (Fig. 2B).

### Effects of *M. persicae* Infestation Duration, Density, Lag Duration after Feeding and Systemic Responses on *B. tabaci* preference

#### Direct effect experiment

**Figure 3:**
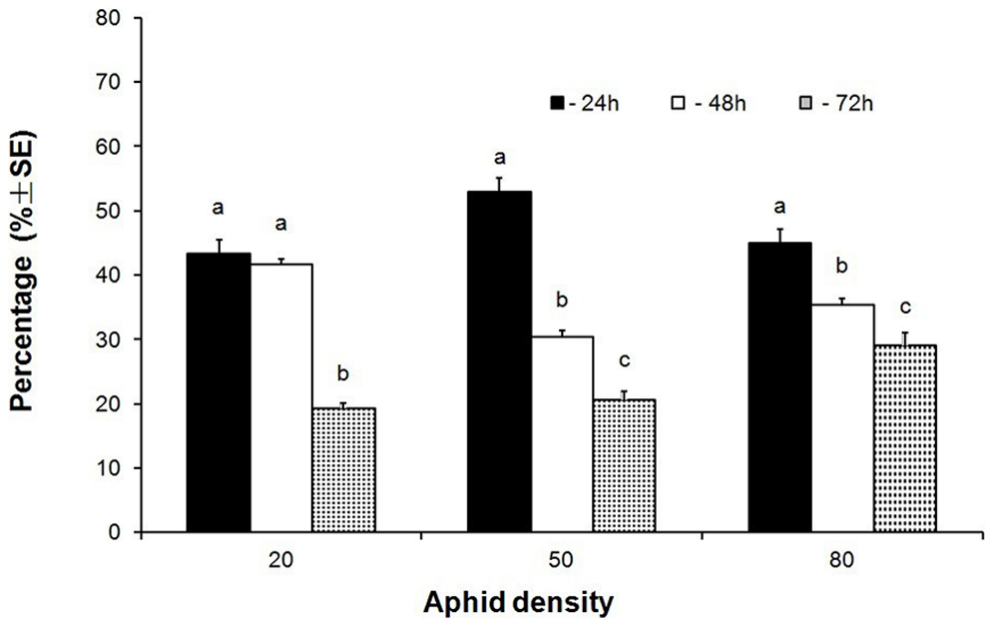
Mean percentages (±SE) of *B. tabaci* adults on the leaves with multiple treatments (duration of aphid infestation and aphid density) as direct effect. Means with different letter were significantly different at *P* = 0.05 (Fisher's positive Tukey test).

**Figure 4:**
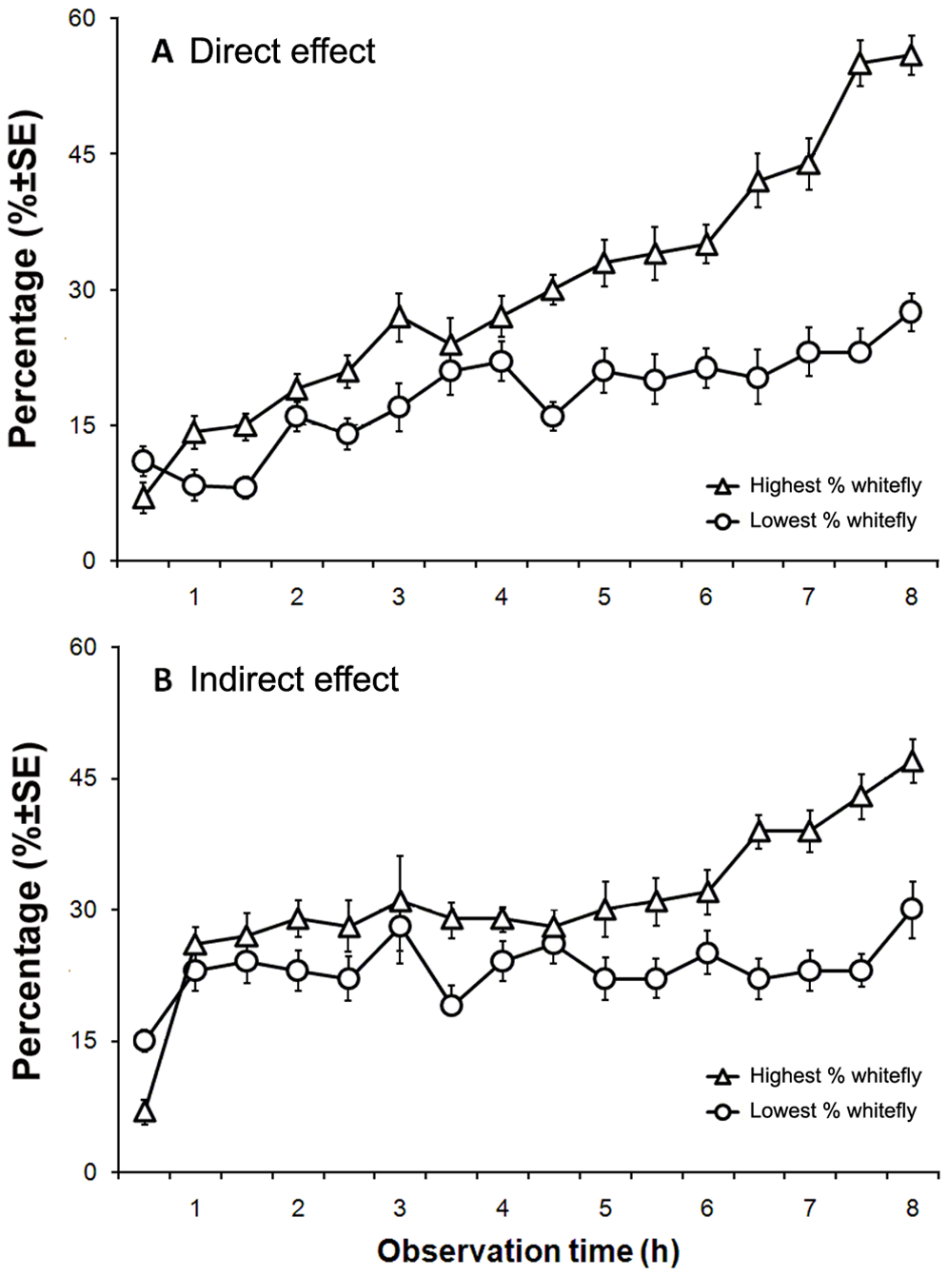
Mean percentages (+SE) of *B. tabaci* adults on treated leaves over different observation times in the four treatments from direct and indirect orthogonal design tests. A. The lowest whitefly percentage treatment and highest whitefly percentage treatment in the direct effect tests (with aphids on the leaves); B. The lowest whitefly percentage treatment and highest whitefly percentage treatment in the indirect effect treatments (after the aphids were removed).

In Exp. 5, the two factors, duration of aphid infestation and aphid density, showed different effects on *B. tabaci* adult preference (Fig. 3). The duration of aphid infestation significantly affected the preference of whitefly adults (*F* = 544.89; df = 2, 351; *P*<0.01), but the aphid densities did not (*F* = 0.98; df = 2,351; *P* = 0.38>0.01). However, there was a significant interaction effect on whitefly preference between the duration of aphid infestation and aphid density together (*F* = 35.70; df = 4, 351; *P*<0.01). Aphids at 50 aphids/leaf and infested for 24 h had a significant positive effect on whitefly preference, and the treatment with 20 aphids/leaf and infested for 72 h significantly reduced whitefly preference. Again, the percentages of whitefly adults landed on the leaves increased over time (Fig. 4A).

#### Indirect effect experiment

In Exp. 6, all four factors showed significant influences to whitefly preference (Infestation duration: *F* = 60.51; df = 2,351; *P*<0.01; Aphid densities: *F* = 51.19; df = 2,351; *P*<0.01; lag duration: *F* = 124.7; df = 2,351; *P* = 0.008; leaf position: *F* = 10.23; df = 2,351; *P* = 0.003) (Table 3). The longer duration of infestation, the lower the percentages of whiteflies on the plants. The 20 and 50 aphids/leaf treatment caused similar effects on whitefly preference, which were greater than that with 80 aphids/leaf treatment. The treatment of 48 h after aphid removal had more whiteflies than in the 0 and 24 h treatments. The leaf with aphids had fewer whiteflies than those on the leaves below and above. Over all the treatments, the one with the lowest response by whiteflies was 72 h of infestation, 80 aphids/leaf, 24 h of time interval after aphid removal, and on the leaf with aphids; while the highest response by whiteflies was 24 h of infestation, 50 aphids/leaf, 48 h of time interval after aphid removal, and the leaf below the aphid-infested leaf. The percentage of whiteflies landing on the leaves increased over time in the treatment attracting the most whiteflies (highest whitefly response), but not in the treatment attracting the fewest whiteflies (lowest whitefly response). The percentage of whiteflies did not increase proportionally with the time and always fluctuated up and down around the average in the treatment attracting the fewest amount of whiteflies (lowest whitefly response), and some of the whiteflies that landed in the plant flew away (Fig. 4B).

#### The highest and lowest responses of *B. tabaci* in the direct and indirect treatments with aphid infestation against a control plant with no aphid infestation

**Figure 5:**
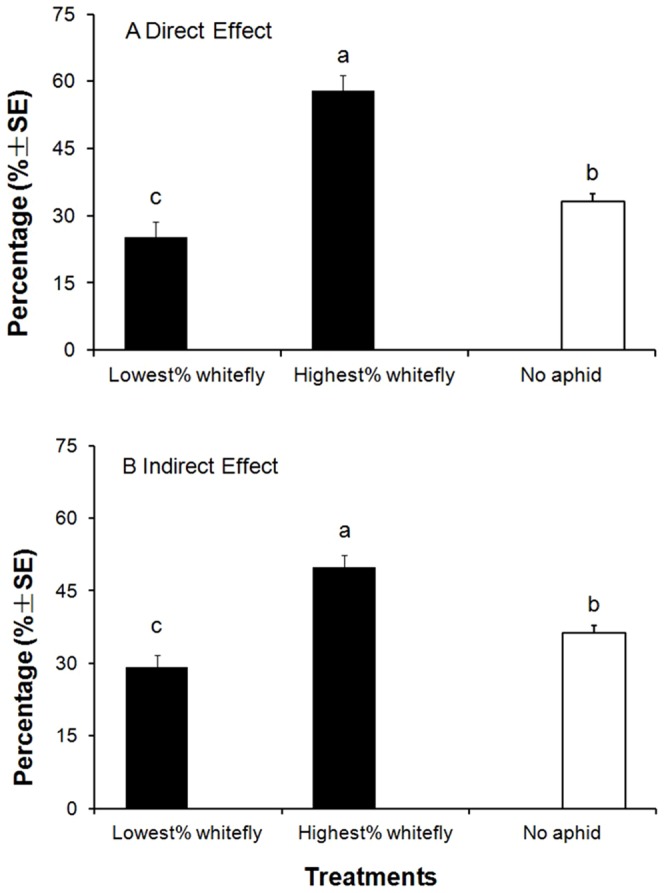
Mean percentages (±SE) of *B. tabaci* adults selective proportions on highest, lowest selected treatments and blank control plant (CK). A. the selective proportion of *B. tabaci* in direct influence experiments; B. Proportion of *B. tabaci* in indirect influence experiments. The same letters inside the columns means that the means were not significantly different at *P* = 0.05 (Fisher's positive Tukey test).

In Exp. 7 for the direct effect experiment, the three treatments had the highest whitefly responses, the lowest whitefly responses and the control treatment. The treatment with the highest whitefly response again had the highest percentage of whiteflies, and the treatment with the lowest whitefly response had the lowest percentage of whiteflies. But the control treatment response was significantly different from both of these and in between (*F* = 15.73, df = 2,117; *P*<0.01) (Fig. 5A). In Exp. 7 for the indirect effect experiment, the three treatments were the highest whitefly response, the lowest whitefly response and the control treatment. The results were similar to those in the direct effect experiment, with the control treatment in between the highest and lowest responses and significantly different from both of them (*F* = 9.14, df = 2,117; *P*<0.01) (Fig. 5B).

## Discussion

Interspecific interactions between herbivorous arthropods which share the same host plants have been widely documented [39]–[43]. Our observations indicate that *M. persicae* influenced the responses of *B. tabaci* adults to tomato plants in both direct effects when aphids were present and indirect effects on plants preinfested by the aphids. Furthermore, *B. tabaci* responses were influenced by several spatial and temporal aspects of the aphid infestation.

It has been reported that aphids have both positive and negative interactions with other plant phloem consumers via plant responses [44]. Aphids penetrate the leaf of the host plant through their stylets and induce plant responses through plant hormone regulation, defensive protein expression and secondary metabolite emission[45]–[47]. Our results showed that few *B. tabaci* were found on the host plant with *M. persicae* when they had a choice of plants with no aphids (Exp. 1). We observed that some whiteflies did land on the host plants with aphids and left immediately or within a short period of time from a few seconds to less than a minute. The presence of aphids having physical contact with whiteflies [6], [48], may directly complete with them for space and food resources, produce alarm pheromones when were disturbed by the competitors [46]. In the direct-choice effect experiment (Exp. 1), despite the whiteflies were directly interfered by the aphids, the defense of the plants infested by aphids had been induced before the release of whiteflies. Therefore the direct effect may be in fact a combination of both direct and indirect effects, although the direct effect when aphid existed on the plant played a more dominant role than the indirect effect. By reviewing the video footage, we found that *M. persicae* did not immediately disturb the landing of *B. tabaci*, and that aphids moved around on the leaves, occupying the space where the whiteflies were present. Meanwhile, whiteflies move also around once they landed on the plant leaves. Although aphids did not inflict severe body damage with physical attack as other aggressive aphids (e.g. Hormaohididae and Pemphigidae species), some *M. persicae* disturbed recently landed whiteflies by kicking them with their legs.

We observed that the plants with aphids had more whiteflies than of the plants with no aphids in the no-choice tests (Exp. 2). A reasonable explanation is that the volatile emitted by the plant infested with the aphid can impact their neighbour plant without aphid attacking [49], [50], and the plants may become more attractive to the whitefly in the choice test. In the no-choice tests, the control plant with no aphids was isolated such that it could not affect the plant infested with aphid. This meant that the plants preinfested with aphids attracted more whiteflies than the plants with no aphids, for certain aphid densities, and feeding durations. In the direct-no choice effect test (Exp. 2), the observed attraction effect to whiteflies suggest that the indirect effect played a leading role when the whiteflies did not have a choice of the plants that was not infested by aphids. Aphid feeding may induce transcription of plant hormones, such as jasmonic acid and salicylic acid, which increase the resistance of host plants to *B. tabaci* [51]. and increase the attraction of aphids [52]. It has been found that the feeding of the Russian wheat aphid, *Diuraphis noxia* (Kurdjumov), enhances the level of nutrition and attractiveness of host plant to other aphids [53]. In contrast, the infestation of *M. persicae* on potato plants did not have a significant effect on *Macrosiphum euphorbiae* (Thomas) [54]. However, *M. euphorbiae* damage increase the attractiveness to *Spodoptera exigua* (Hübner) on tomato plant [55]. It appears that the preference of whitefly is not only limited by the arriving sequence of herbivore insects, plant suitability and characteristics, but may also benefit from conspecific feeding via modification and increasing of environmental stress [7], [48]. Our results showed high sensitivity of the whitefly to pre-infestation by aphids, even though few previous works demonstrate that whitefly was more sensitive to aphid's preinfestation compared to other herbivores [55].

Our results showed the negative impacts of *M. persicae* infestation were different between choice and no-choice treatments in the indirect effect experiments (Exp. 3). These impacts may imply a state of continuous piercing-sucking modification of aphids to tomato plant and direct effects on whiteflies response when the aphids still existed. After interacting with the aphids, the whiteflies may gradually become accustomed to with the presence of aphids. The whitefly's response was negatively affected by the plants with previous aphid infestation when the whitefly have no a choice of a tomato plant without infested by aphids (Exp. 4). But the percentage of whiteflies increased significantly more than that of in the treatment of whitefly having a choice of uninfested tomato plant or in the treatment of aphids existed. This indicated that the plants infested by aphids deterred whiteflies from landing, but over time the deterrence was gradually lessened, probably because the aphids can not feed continually, or may be due to the plants that release volatile organic compounds attracting the whiteflies. The whiteflies were able to settle on plants which aphids previously infested, and were able to settle on the leaf with aphids if no uninfested plants were available. These results showed that the defensive response induced by the aphids was short-lasting, and suggested that the plant mediated interaction between herbivores could be promoted once the induced defense had been triggered [56].

Our results from the direct effect and indirect effect treatments in the orthogonal design experiment showed that the inhibitions of aphid infestation to *B. tabaci* preference were strongly enhanced with the prolonging of the duration of aphid infestation. It is intuitive that the longer infestation duration of aphids on the plant results in stronger deterrence to newcomers because induction of the plants defensive system takes some time [57]. We found that the aphid densities had different impacts on the response of *B. tabaci* depending on the duration of aphids presence and removal. One possible explanation is that the efficiency of *B. tabaci* inhibition was primarily determined by the infestation duration of the aphids. Early research on the cereal aphid, *Rhopalosiphum padi* (L.), on wheat, *Triticum aestivum* L., show that lower aphid density does not increase the amount of defensive hydroxamic acids [57]. However, as shown in our data, when the aphids were removed, the inhibitive effects to *B. tabaci* were enhanced with the increasing of aphid density and decreasing of time interval after the aphids were removed. These results indicate that the induced resistance by the plant might be short-term, which means the optimal time for preventing arrival of other species could correspond to the time of aphids removal. This kind of effect could be attributed to the composite action of the defense enzymes and the volatile organic materials [17], [47]. In the treatment attracting the fewest whiteflies of direct effect experiment, the arrival pattern of whitefly was wave-like, with some whiteflies subsequently leaving the plant. Further, the number of whiteflies was stable until the four hour mark, which indicate that induced defense has instantaneous impact (Exp. 5). In the indirect effect experiment, the pattern of the lowest response by whiteflies (Exp. 6) did not increase proportionally with the time, whereas the highest response did. This shows that the treatment leaves with high aphid density (50 aphids/leaf), highest feeding duration and short time lag are the most effective in deterring whiteflies even over the 8 h of the original observations. Further, different from the treatment attracting the fewest whiteflies in direct effect experiment, the average number of whiteflies not stable until the last hour. This indicates that in addition to the emission of volatile organic compounds, non-volatile organic compounds contribute to repelling whiteflies. In the treatment attracting the highest amount of whiteflies, the number of whitefly landing on the plant gradually increased over the assessment period until the 6th or 7th hour at which time the number of whitefly rapidly increased. This shows some persistence of the defense reaction. When the time interval between removing the aphids and releasing whiteflies increased to ≈48 h, the effect of plants infested by aphids which deterred whiteflies decreased. Our observations showed that *B. tabaci* was strongly inhibited by the volatile blends in the treatments in which the aphid removal to whitefly release intervals was shorter, with an interval of 0–24 h, optimal for producing the resistance. It has been reported that when the feeding duration is shorter, more time is required before release of some herbivore-induced plant volatiles (HIPVs), after which their emission rate increases for several hours [58], [59]. Some compounds including terpenoids, are synthesized again and released from several hours to several days after attack [58]. Understanding the interactions between early and late arriving herbivores via plant-mediated defensive responses should help us to understand natural mechanisms for management of herbivore pests, and to develop strategies to enhance natural control processes of agroecosystems. We already know that the emission of many HIPVs is relative to factors such as the feeding duration, herbivore density, and the interval duration. Through comparison of the highest and lowest responses by *B. tabaci* in leaves with aphid infestations against a control plant with no aphid infestation, it is apparent that the influence of tomato plant with different aphid feeding duration, density, lag duration and leaf position on *B. tabaci* varied with level of attraction or deterrence.

Herbivore feeding may cause systemically defensive responses requiring synergistic contribution of various tissues of the host plant [60], [61]. The induced defenses may be originated in undamaged parts (e.g. leaves or roots) of the herbivore-attacked host plant, which has been widely documented in previous studies [17]. Our studies also suggest a systemic response to *B. tabaci* induced by *M. persicae* among the three observed leaves, with more *B. tabaci* on the aphid-infested leaf than on the leaves at lower and higher position which lacked aphid infestation. However, whiteflies were attracted by the lower and higher tomato leaves, especially the lower leaf. Maybe the volatile organic compounds (VOCs) released in the lower leaf which was more attractive to the whiteflies. This indicates systemic response occurred among the three different leaves. The molecular mechanism behind systemic defense consists of expression of protective proteins, generation and accumulation of defensive volatile organic compounds, along with reciprocity between plant tissues [8], [12], [16], [62]. It has been reported that the resistance of tomato plants to *B. tabaci* was both locally (LAR) and systemically (SAR) expressed [48], [63]. Xue *et al.* [6] found that *B. tabaci* feeding on lower or older leaves of the tobacco caused systemic defense on the upper and younger leaves, which influenced the overall fitness of *M. persicae*. It has also been found that feeding damage by some insects results in long-distance transport of signal molecules that may elicit changes in distant leaves [64], [65]. Some studies showed that whitefly infestation elicited defensive response signaling in both upward and downward directions [66]. Similarly, it has been reported that *S. exigua* gradually moved down the plant with previously damaged to feed on the older leaves [67], which had similar results to ours. One possible reason is the evading of natural enemies attracted by volatile defensive material from the damaged host plant [7].

Although the defensive chemicals cannot be easily separated from aphid feeding or mechanical damage, previous researches have revealed that plants can differentiate between mechanical wounding and damage caused by herbivore insects because mechanically damaged or healthy plants generally do not produce or produce only small amount of terpenoid substances [68], [69]. In contrast, plants release a large number of terpenoid chemicals after they are infested by herbivores [58], [59]. When herbivores feed on plants, the specific compounds in the insect's oral secretion activate and trigger the emission of VOCs or attractive odors to attract natural enemies [70]. For instance, mechanical damaged corn seedlings do not produce much terpenoids, but the insect damaged corn emits a large amount of larvae feeding related terpenoid substances [70], [71]. At the transcriptional level, potato mRNAs involved in plant defense accumulate more rapidly with insect-derived elicitor(s) in contact with the damaged leaves than with mechanical damage alone [72].

In conclusion, infestation by the phloem sap probing aphid *M. persicae* directly and indirectly impacted *B. tabaci* preference to local and systemic leaves. The results also indicate that duration of infestation by *M. persicae* was a key influential factor on whitefly preference; and aphid density was another important factor in the indirect effect on the whitefly after the aphids were removed. Long infestation period (72 h) with high aphid density (80 aphids/leaf) should be more efficient to defend against incoming whiteflies. The current research demonstrated the specific induction and effects of systemic resistance of plant, which will contribute to the understanding of complicated plant-herbivore-invasive herbivore colonizer interactions.
